# Feasibility and acceptability of a solution-focused approach to strengthen lay counselling for common mental disorders (DIALOG+) in Pakistan: mixed methods study

**DOI:** 10.1192/bji.2025.4

**Published:** 2025-08

**Authors:** Saniya Saleem, Anayat Baig, Onaiza Qureshi, Sana Sajun, Victoria Bird, Stefan Priebe, Aneeta Pasha

**Affiliations:** 1 Senior Manager, Mental Health, Interactive Research and Development, Karachi, Pakistan. Email: onaiza.qureshi@ird.global; 2 Program Associate, Mental Health, Interactive Research and Development, Karachi, Pakistan; 3 Project Manager, Unit for Social and Community Psychiatry, WHO Collaborating Centre for Mental Health Services Development, Queen Mary University of London, London, UK; 4 Director, Unit for Social and Community Psychiatry, WHO Collaborating Centre for Mental Health Services Development, Queen Mary University of London, London, UK; 5 Director, Unit for Social and Community Psychiatry, WHO Collaborating Centre for Mental Health Services Development, Queen Mary University of London, London, UK; 6 Director, Mental Health, Interactive Research and Development, Karachi, Pakistan

**Keywords:** Common mental disorders, solution-focused approach, mental health, community care, DIALOG+

## Abstract

**Background:**

Depression and anxiety are widespread globally, with significant treatment gaps in low-resource settings. In Pakistan, where prevalence is high and specialists are scarce, brief psychological interventions by trained lay counsellors show promise. DIALOG+ is a novel technology-assisted, solution-focused approach for leveraging resource-oriented approaches in routine community mental health treatment.

**Aims:**

To explore the feasibility and acceptability of using DIALOG+ for community-based treatment of common mental disorders delivered by non-specialist lay counsellors in a low-resource setting (trial registration: ISRCTN14528579).

**Method:**

An open, uncontrolled trial in community settings in Karachi, Pakistan, was conducted with 40 patients with depression and anxiety visiting two primary care clinics between June 2019 and February 2020. Patients were enrolled for monthly sessions delivered over 6 months by lay counsellors. Subjective quality of life along with symptoms of depression and anxiety were measured at baseline and endline (following the 6-month intervention) on the Manchester Short Assessment of Quality of Life (MANSA) and Aga Khan University Anxiety and Depression Scale (AKUADS). Changes in measures were evaluated before and after the intervention using a *t*-test analysis. Post-intervention, in-depth interviews were held with patients and lay counsellors to gather insights into their experience of the intervention.

**Results:**

In total, 146 DIALOG+ sessions were conducted with 40 patients. At the 6-month post-intervention assessment, 33 patients showed improved subjective quality of life and reduced self-reported depression and anxiety scores. Patients reported that the intervention helped strengthen the therapeutic relationship with their lay counsellors, helped them track their progress through therapy and enhanced their self-management of negative emotions and behaviours.

**Conclusions:**

Structured communication can help strengthen lay counsellors’ ability to improve therapeutic outcomes of people with common mental disorders in resource-constrained community settings. Future clinical trials are recommended to further evaluate the long-term impact of the DIALOG+ intervention on mental health outcomes.

Globally, over 300 million people are living with depression and anxiety disorders, making them major contributors to disability.^1^ These common mental disorders can have a negative impact on an individual's physical health, quality of life, social relationships and participation in community and economic opportunities.^2^ Brief psychological interventions are effective as first-line treatments for these disorders and are supported by the World Health Organization's Mental Health Gap Action Programme (mhGAP).^3^ It is estimated that nearly 90% of people with common mental disorders struggle to receive appropriate care.^4^ Evidence suggests that first-line psychosocial counselling approaches delivered by trained and supervised lay counsellors (non-specialist community mental health counsellors) through a stepped-care model in healthcare and community settings are effective in reducing symptoms and addressing the treatment gap in mental health support in resource-limited contexts.^5,6^

In Pakistan, studies exploring the prevalence of depression and anxiety report rates between 22 and 60%.^7,8^ There are limited mental health professionals to cater to this need: for example the country has only 1 psychiatrist per 500 000 people.^9^ This treatment gap is largely attributed to the shortage of mental health resources, especially in remote and low-income areas.^10^ Evidence-based mental health interventions have long recommended task-shifting as a way to redress the treatment gap in low- and middle-income countries.^11,12^ Task-shifting models allow trained lay counsellors to provide front-line mental health support for common mental disorders, while referring patients with more severe disorders to specialist providers (such as psychiatrists and psychologists).

Resource-oriented approaches are those that help patients better utilise existing resources and social support networks to alleviate distress.^13^ These approaches can be delivered in diverse settings, without the need for additional costs, specialist mental health providers or advanced clinical training. Moreover, identifying and evaluating such resource-oriented mental health approaches that can be delivered by lay counsellors is a priority in the global mental health field.^14^

## DIALOG+

DIALOG+ is a brief, technology-assisted approach designed to structure routine clinical consultations between patients and mental health professionals.^15^ The intervention is delivered using a tablet or smartphone application (app) and is grounded in principles of patient-centred care and solution-focused therapy. In DIALOG+ sessions, patients use the app to assess their satisfaction across eight holistic life domains and three treatment domains. Following this assessment, a structured four-step solution-focused approach is proposed to target low satisfaction in specific domains by identifying personal resources and developing strategies to address their concerns.^15^ Lay counsellors can benefit from utilising a guided approach such as DIALOG+ to improve the structure of their counselling sessions and provide more complex patient-centred support. In high-burden settings with a risk of provider burnout, a standardised approach can facilitate effective decision-making to ensure that patients are provided quality support in target areas of concern without contributing to additional fatigue.^16^ Trials conducted on DIALOG+ have shown it to be effective at enhancing quality of life and treatment outcomes for people with severe mental illness in high-resource settings.^17^ However, it has not been used with patients with common mental disorders.

## The current study

This study aims to test the feasibility, acceptability and effectiveness of implementing the DIALOG+ intervention for people with depression and anxiety in Pakistan. The objectives of this study are to explore:

- the impact of DIALOG+ for improving quality of life, mental health and social outcomes for patients with symptoms of depression and anxiety;
- how the intervention is experienced by patients and their lay counsellors;
- how DIALOG+ can be used to strengthen community mental healthcare in Pakistan.

## Method

### Design

We conducted an open, uncontrolled trial at the Indus Health Network's (IHN) two primary care clinics with integrated mental health services, serving low-income communities free of charge in Karachi, Pakistan. Patients visiting the primary care centre were assessed for depression and anxiety and enrolled for a brief intervention by a lay counsellor utilising a task-sharing approach.

### Sample selection

We used purposive sampling to recruit into the study 4 lay counsellors already employed by the Interactive Research & Development (IRD) Mental Health Program and open convenience sampling to recruit 40 patients attending the IHN primary care clinics. Given that this is an exploratory study, a smaller sample size is often adequate to provide initial insights and identify trends or patterns that can inform larger, more definitive studies.^18^

A research associate (A.B.) assessed lay counsellors for eligibility to participate in the study. Lay counsellors were eligible if they had received basic counselling training by psychologists working in the IRD Mental Health Program, had more than 6 months’ experience of mental health screening and lay counselling of patients and had no plans to leave their job during the study period.

Lay counsellors were based at the IHN primary care clinics and conducted the eligibility assessment of patients with symptoms of depression and anxiety at the primary care clinics. Consecutive patients visiting the IHN primary care clinics were screened for eligibility until the enrolment was complete. Patients were invited to participate in the study if they met the following criteria:

- aged between 18–65 years
- resided within 20 km of the IHN primary care clinics
- had symptoms of depression and anxiety as reported by a cut-off score of ≥20 on the Aga Khan University Depression and Anxiety Scale (AKUADS).

Patients were excluded if they:

- had a mean score of ≥5 on the Manchester Short Assessment of Quality of Life (MANSA) scale
- did not speak Urdu or English
- could not give informed consent.

### Data instruments

This study employed a mixed methods approach, utilising both quantitative and qualitative data collection and analysis. Demographic information was collected for all participants, including age, gender, marital status, educational attainment and employment status.

The primary outcome measure was subjective quality of life, assessed using the Manchester Short Assessment of Quality of Life (MANSA). The MANSA consists of 16 items, with participants rating their satisfaction across 12 life domains on a Likert scale ranging from 1 (totally dissatisfied) to 7 (totally satisfied).^19^

Secondary outcome measures included depression and anxiety symptomology, as well as social outcomes. The Aga Khan University Depression and Anxiety Scale (AKUADS) is a 25-item screening tool designed and developed locally to assess symptoms of depression and anxiety in Pakistani populations.^20^ Each item is rated on a 0–3 scale, yielding scores ranging from 0 to 75, with higher values reflecting a greater severity of symptoms. A cut-off score of ≥20 suggests the probable presence of depression and/or anxiety symptoms. The Social Outcome Index (SIX)^21^ serves as an objective indicator of social functioning, evaluating factors such as employment status, accommodation and social relationships of individuals with mental illness. Scores range from 0 to 6, with higher scores indicating more positive social outcomes.

A topic guide was created in Urdu and utilised for semi-structured interviews with a selected group of patients and counsellors. The aim of this was to understand their experience of DIALOG+, related challenges and facilitators to attending sessions, gathering suggestions for potential adaptations and its implementation in the local context.

Data from the DIALOG+ app were extracted to analyse the action items agreed between the patient and the lay counsellors at the end of each session.

### Ethics approval

Ethics clearance was granted by the Interactive Research Institutional Review Board (IRB 00005148) and Queen Mary University London's Ethics of Research Committee (QMERC 2019/21). The trial was registered on 6 June 2019 (ISRCTN14528579).

### Procedure

Participants were recruited between 12 June 2019 – 4 September 2019 and follow-up assessments were conducted between 14 December 2019 and 22 February 2020. Recruitment was conducted after obtaining written informed consent from all participants.

All lay counsellors participating in the study received a 2-day training on the principles of DIALOG+ and delivery of the intervention using an android tablet. Counsellor and patient met monthly over a 6-month period, with each meeting lasting 20–60 min. During each meeting, patients used the DIALOG+ app to rate their satisfaction across eight life domains and three treatment-related domains. Ratings were displayed visually on the app for current and prior sessions, allowing for review of changes in satisfaction rates and patient progress. Patients then selected which domains they wished to focus on during the regular meetings. Each chosen domain was addressed through a structured four-step approach which involved: (a) ‘understanding’ or identifying the reasons for dissatisfaction and acknowledging what has been working; (b) ‘looking forward’ or exploring the best possible outcomes and the smallest action required for improvement; (c) ‘exploring options’, where potential actions that patients, lay counsellors and others could take for improving satisfaction in a domain are detailed; and (c) ‘agreeing on actions’, where both patient and lay counsellors decided on concrete steps to pursue before the next session. The agreed actions were subsequently documented as action points and reviewed to track progress during the next meeting.

### Data analysis

Quantitative data were analysed using Open Access R software (version 3.6 for Windows). Demographic variables were reported with means and standard deviations for numerical data and frequencies for categorical data. Paired *t*-test was used to compare the change in outcome measures over time, along with confidence intervals. *P* < 0.05 was considered statistically significant. Owing to the small sample size, missing data were not addressed in the analysis, given the exploratory nature of the study, where the primary focus was on generating preliminary insights rather than reaching definitive conclusions.

Qualitative interviews were conducted in Urdu and transcribed verbatim. An inductive analysis approach with open coding was used to generate unique insights and in-depth understanding of interviews. Two research team members familiarised themselves with the transcripts and then conducted open coding with similar codes grouped under themes. This was followed by checking and refining the identified themes and sub-themes. Content analysis of the action items recorded in the sessions was also conducted to explore the nature of the action item and the individual (patient, lay counsellor or other) responsible for the agreed action item.

## Results

**Fig. 1:**
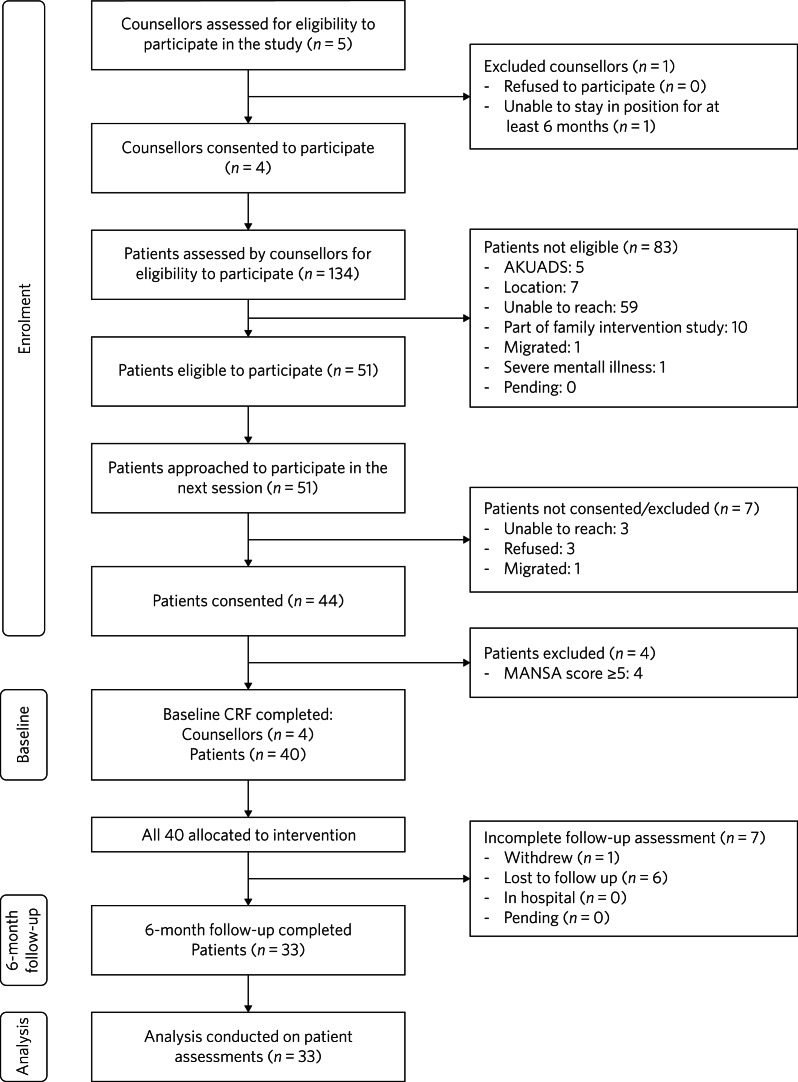
CONSORT flowchart of study participants (patients and counsellors) for the uncontrolled open trial. AKUADS, Aga Khan University Anxiety and Depression Scale; CRF, case report form; MANSA, Manchester Short Assessment of Quality of Life.

Total of 5 lay counsellors and 134 of their patients were assessed for eligibility. Following the screening, 4 lay counsellors and 40 patients were enrolled to participate in the intervention. The 6-month follow-up and subsequent analysis was conducted with 33 (82.5%) of the enrolled patients (Fig. 1).

Patients were predominantly female (80%) and married (68%), with an average age of 34 years. Two of the counsellors were female and two were male (Table 1).

**Table 1:** Demographic characteristics of patients and counsellors enrolled in the study

Characteristic	Category	Patients (*n* = 40)		Lay counsellors (*n* = 4)	
		*n*	%	*n*	%
Age, years	18–25	9	23	2	50
	26–35	14	35	2	50
	36–45	12	30		
	46–55	5	13		
Gender	Female	32	80	2	50
	Male	8	20	2	50
Marital status	Married	27	68	1	25
	Single	10	25	3	75
	Separated or Divorced	3	8		
Ethnicity	Urdu-speaking	23	58	3	75
	Pushto	7	18		
	Siraiki	3	8		
	Other	7	18	1	25
Education	Primary education or less	13	33		0
	Secondary education	13	33	3	75
	Tertiary/further education	14	35	1	25

A total of 146 sessions were delivered to 40 patients. Overall, the patients participated in an average of 3.65 sessions (s.d. = 1.64) over 6 months. Of the 40 patients, 27 (68%) completed at least 3 sessions, with an average of 4.62 sessions (s.d. = 0.94). Six-month assessments were completed for 33 patients (83%). Paired comparisons of subjective outcomes are presented in Table 2. Frequency distributions of objective social outcomes pre- and post-intervention are presented in Table 3.

**Table 2:** Changes in mean subjective quality of life, anxiety and depression and objective social outcomes before and after the intervention

Tools	Baseline (*n* = 40), mean (s.d.)	6 months (*n* = 33), mean (s.d.)	*t*	95% CI	Cohen's *d*
Subjective quality of life (MANSA)	3.73 (0.68)	5.11 (0.83)	7.7**	1.03–1.74	1.82
Anxiety and depression (AKUADS)	32.22 (7.52)	17.23 (7.20)	8.39**	11.05–17.94	2.04
Objective social outcomes (SIX)	3.73 (1.48)	4.48 (0.91)	2.7*	0.20–1.32	0.61

MANSA, Manchester Short Assessment of Quality of Life; AKUADS, Aga Khan University Anxiety and Depression Scale; SIX, Social Outcome Index.

* *P* *<* 0.05, **P *<* 0.001.

**Table 3:** Frequency distribution of employment, accommodation and social outcomes pre- and post-intervention

Variables	Baseline (*n* = 40)		Six-month (*n* = 33)	
	*n*	%	*n*	%
Employment				
No employment	31	78	21	64
Voluntary	−	−	1	3
Regular employment	9	23	11	33
Accommodation				
Homeless	3	8	−	−
Supported housing	−	−	−	−
Private housing	37	93	33	100
Partnership/family				
Living alone	3	8	1	3
Living with partner/family	37	93	32	97
Friendship				
Did not meet a friend in the last week	21	53	6	18
Met a friend in the last week	19	48	27	82

### Content of DIALOG+ sessions

Across a total of 146 sessions, an average of 1.4 (s.d. = 0.6) domains were discussed in each session. The most prevalent domains discussed were mental health, physical health and job situation.

In total, 327 unique action items were recorded, with an average 2.53 items discussed per session (Table 4). Patients were assigned as the primary person responsible for implementing 276/327 action items (84%), followed by lay counsellors at 43/327 (13%) and ‘others’ such as family and friends at 8/327 (2%). Patient-led actions included actions towards a healthier lifestyle (e.g. exercise, diet, medication management), seeking support from someone and practising self-care and coping strategies. The majority of counsellor-led actions related to providing directive advice.

**Table 4:** Action items (*n* = 327) discussed in DIALOG+ sessions

Action items	*n*	%
Patient	276	84
Healthier lifestyle (nutrition, self-care)	84	
Coping strategies (spiritual, activities, meditation)	75	
Talk to someone/seek social support	46	
Practical actions (talk to clinician, change environment, seek employment)	71	
Counsellor	43	13
Listen and give advice	38	
Support job search	4	
Speak to family member	1	
Others	8	2
Provide social and/or financial support	7	
Improve environment	1	

### Views of patients and counsellors

There were 12 individual interviews conducted (8 with patients; 4 with counsellors). Four themes emerged regarding the use of DIALOG+ in the current setting: (a) the therapeutic relationship; (b) tracking progress; (c) self-management; and (d) novel experience. An outline of these themes, with quotations for illustration, are presented in Table 5.

**Table 5:** Themes from in-depth interviews with patients and lay counsellors post-intervention

Themes	Clarification and quotations
Strengthening of therapeutic relationship	This theme relates to the impact of DIALOG+ on the relationship between the counsellor and the patient. Counsellors reported that they experienced the sessions and planning actions as a collaboration with the patients instead of directed by them, and patients highlighted the guidance that they received from their counsellors: ‘They [patients] knew what their goals were and how they could achieve them, so in this [before] what the counsellor would do, in this there was mutual sharing. So building trust did not take long, it developed quickly’ (C3) Patients also reported the strong kinship that they felt with the counsellors, and the counsellors’ role in helping them navigate and deal with their stressors: ‘I was really disturbed when I came to him […] he made a roadmap for me on how to deal with my challenges, and because of this I am able to easily deal now’ (P26) ‘I think we have developed a very good friendship […] she understood me, my problems […] I am following her guidance till today’ (P16)
Tracking progress	Counsellors and patients noted that the ability to visually track progress through the treatment period: ‘When the patient rated between 1 to 7, we got to know what stage s/he were at and helped us understand them better. And even the patient when they saw last time's [rating] they would feel that yes there is an improvement, and when there was no improvement they thought they should try to move ahead […] this was helpful’ (C2) ‘When we asked them how they would rate this [domain] today, they would ask what did I rate this the last time […] this made it easy for them […] They wanted to see improvement’ (C3) ‘The best thing was the patient could see themself where they stood, how they ranked and where they want to be and how to get there’ (C3) ‘I would go every month and some months there was a change and through the numbers [rating] some months something good happened and I could give more numbers and some months I would give lower numbers […] and the points that were less have increased every month. I am at a 6!’ (P25)
Self-management	Counsellors and patients reported a noticeable change in the patient's self-management of emotions and behaviours, by focusing on the positive: ‘Initially when I was going he [my husband] said that this will not do anything but when he saw the change in me he promoted me to go, this is the first time I have left the house myself, there is more confidence in me’ (P16) ‘I would not do any work before, my heart was not in it, now I do work. When she [my counsellor] asked me about my friends, I told [her] I didn't make any, did not even try, only my husband is my friend. Now I go and meet people’ (P40) ‘When I came to him [my counsellor] […] I told him in my heart I wanted to end my life and I really wanted to, I even wanted to leave my daughter at that time, I wanted to end my life and take her with me, I didn't want her to experience the same worries I did […] now I feel like doing something, before I didn't want to do anything, just felt like everything was ending’ (P25) ‘Like my anger from before is much less, like the capacity to tolerate was less, now it's more […] my family also felt it […] Using the app at least helped me control my anger so I am quite satisfied […] my husband and father-in-law also noticed a change in me, it felt good’ (P16) ‘I felt self-doubt before but since my attitude started changing, the same things I start to see positively’ (P26) ‘There were improvements, energy levels increased, and people got involved in various activities, the goals they set they were also able to achieve’ (C3)
Novel experience	Counsellors and patients identified the experience as a new learning opportunity: ‘We are also learning something or the other from the patient. Because everyone experiences stress in their daily life so we also get to learn a lot from them. When we talked about actions the patient would be forced to think; for example someone thinks I need a job, so when we make action plan for what they can do, where all they can apply, from there they think and their thoughts turn towards the positive and we both benefit from it’ (C4) ‘Learnt of a lot of new things, things that I never even thought of or know of, that were not even in my mind’ (P16) Counsellors and patients did note that literacy levels may affect the use of the technological intervention in the current context: ‘You are telling your heart's state […] in the app you feel like you are doing some technical, official work, the patient can get affected, especially a common man who can't understand these things may hesitate, maybe he wouldn't know what will happen to him’ (P26) ‘An educated person could easily use this at home, teachers could use it in offices, where people are more literate’ (C2)

## Discussion

This study explored factors related to the implementation of DIALOG+ as a low-cost technological approach for strengthening community care of patients with anxiety and depression facilitated by non-clinical lay counsellors in resource-constrained settings. We found that the DIALOG+ intervention helped significantly improve subjective quality of life, mental health outcomes and objective social outcomes. Our qualitative findings indicate that DIALOG+ can strengthen the therapeutic relationship between patients and lay counsellors, help patients and their lay counsellors track progress during therapy, enhance patients’ self-management of emotions and behaviours and serve as a novel learning experience for both patients and counsellors.

### Comparison with literature and interpretation of findings

An improvement in subjective as well as objective mental health and social outcomes was seen in patients after 6 months of the intervention. The improvement in anxiety and depression is in line with previous studies which have found that brief psychosocial interventions delivered by trained non-specialist service providers can have a large effect on depression and anxiety outcomes in low resource settings,^5^ with studies reporting intervention effect sizes of 1.3,^22^ 1.2 and 0.7.^23^ This is the first time that DIALOG+ has been implemented for common mental disorders, although previous evaluations of DIALOG+ in the UK with patients with psychosis have reported improvements in subjective quality of life, with medium effect sizes.^16,17^ Although this explorative study reports improvement in clinical outcomes at 6 months, follow-up clinical trials can play a crucial role in assessing sustained improvements in mental health beyond the active period of the DIALOG+ intervention. Most research of brief psychosocial interventions evaluates subjective outcomes of mental health and quality of life and it has been found that subjective and objective measures can differ markedly.^24^ We found that objective social outcomes had also improved at the end of the 6-month intervention period, particularly in the domain of employment and friendship. Our intervention was delivered in a low-income community setting where financial stressors are a major contributor to the burden of mental disorders,^25^ where an improvement in financial situation through employment can help improve subjective mental health outcomes as well.^26^

The communication between patients and their counsellors plays a primary role in mental health outcomes,^27^ and this is supported by qualitative findings in this study. Strengthening therapeutic relationships is associated with patient activation through shared goal-setting and tasks.^28^ The DIALOG+ intervention is based on shared decision-making with the patient, and our findings support a high rate of patient ownership over actions, with a majority of actions being patient-led. This is a shift away from the traditional view of counselling as a directive process with patients seeking guidance, as previous literature indicates that in Asian cultures, patients have indicated a preference for directive counselling, often viewing the service provider as an ‘expert’.^29^ Although the majority of the action items were patient-led, 13% were counsellor-led, indicating the patient's desire for directive advice in problem-solving and highlighting how the preference of patients to participate in their mental health treatment can vary.^30^ This is not surprising considering that the use of talking therapy for mental health concerns in this context is a relatively new area, where the premise and purpose of counselling often need to be explained to the patient. This indicates that patient preferences for participation in shared decision-making may vary and should be accounted for in the treatment approach.

The patient's ability to track their own progress also facilitates engagement with their treatment, enhancing their autonomy in the therapeutic process. A commonly cited reason for attrition from counselling is a lack of congruence between patient and clinician over treatment goals and outcomes.^31^ Through DIALOG+, patients and mental health service providers can visually track patient progress over the course of treatment, which can help manage expectations for both parties about treatment progress as well as serve as motivation for the patient to continue treatment.

### Strengths and limitations

This is the first time DIALOG+ has been used by lay mental health counsellors to treat patients with depression and anxiety. Although the non-randomised uncontrolled trial design allows us to draw only limited conclusions about the effectiveness of the intervention, this design does provide valuable insights into the feasibility and acceptability of implementing DIALOG+ in Pakistan. Another limitation is the small number of participating patients, who were predominantly female. However, that is a reflection of the patient demographic frequenting primary care centres for routine care in this setting. We measured end-line assessment at 6 months, and therefore there is limited knowledge about sustained improvement for long-term outcomes.

### Implications for future research

Our findings indicate that assisting lay counsellors to deliver care through structured communication can enhance the quality of life and mental health outcomes of people in need of support. Further, with minimal additional training and logistical resources, DIALOG+ enhanced counsellors’ confidence in their ability to provide mental health support and strengthened therapeutic relationships. This has important implications for mental health training programmes in low- and middle-income countries where task-shifting models have been promoted as a means of reducing the mental health treatment gap. This study indicates promising findings for proof of concept, and further studies with more rigorous and controlled designs are recommended to evaluate the effectiveness of DIALOG+ compared with treatment as usual provided by lay counsellors. Further, assessing the pathway through which the computer-mediated structured counselling affects mental health outcomes in low-resource settings will help amplify the therapeutic value of the intervention.

## Data Availability

The data collected and analysed in this study are available from the corresponding author (O.Q.) on reasonable request.
